# Introversion and Social Engagement: Scale Validation, Their Interaction, and Positive Association With Self-Esteem

**DOI:** 10.3389/fpsyg.2020.590748

**Published:** 2020-11-30

**Authors:** Sanna Tuovinen, Xin Tang, Katariina Salmela-Aro

**Affiliations:** ^1^Faculty of Educational Sciences, University of Helsinki, Helsinki, Finland; ^2^School of Psychology, Central China Normal University, Wuhan, China

**Keywords:** engagement, student engagement, school engagement, academic engagement, social engagement, well-being, adolescent, high school

## Abstract

Learning through social interaction has been documented widely; however, how introverted people are socially engaged in learning is largely unknown. The aim of this study was, first, to examine the reliability and validity of the social engagement scale among students at Finnish comprehensive schools. Then, we aimed to examine the interaction effect of introversion and social engagement on self-esteem, schoolwork engagement, and school burnout. Based on a sample of 862 ninth grade students in Finland, we found that a two-factor model best fitted the social engagement scale (i.e., social engagement and social disengagement). Further, we found that introverts with high social engagement have higher self-esteem than introverts with low social engagement. Our results implied that introverts should be given extra support when they encounter group work in school.

## Introduction

Learning is a social process in which children gain knowledge through social interaction and exchanging ideas with their classmates (Vygotsky, 1978). Through social interaction, students learn from others, create a positive working environment, provide multiple perspectives, and enhance critical thinking and problem-solving skills (Hurst et al., 2013). Previous research showed also that students who receive support from their classmates are more active at school (Murberg, 2010). In the context of this study, Finland, the importance of good social skills are increasingly emphasized (Jokinen and Sieppi, 2018). Nowadays, many Finnish school tasks include teamwork, collective learning, and discussion-based activities. The new Finnish National Core Curriculum (Opetushallitus, 2016) highlights students' participation in class and conversations, different study environments, new teaching and studying strategies, self-regulation, project-based learning, collaborative learning, and group work.

Despite the prevalent emphasizing of social learning in school, we know little whether these social learning activities may benefit one type of student (e.g., extrovert) but put others (e.g., introverts) at a significant disadvantage. People differ in terms of their inclinations toward the inner and outer world, known as introversion and extraversion, and of the world's population, 30–75% are introverts (Laney, 2002; Helgoe, 2008; Cain, 2013). In general, introverts tend to have low social desire and to withdraw from social activity; thus, it would be important to know how introversion interacts with social engagement in school. More importantly, the understanding that introverts lack social skills are not necessarily true. Many introverts function very well in social situations (Costa and McCrae, 2006), although they might prefer to avoid them due to the overwhelming feeling by too much social engagement (Helgoe, 2008). In other words, introverts can have good social skills, but they may still withdraw from the social activities due to their low social willingness. Therefore, the purpose of this study is to examine how introverts adapt and perform in the social learning situations. Specifically, we aimed to answer the following questions: Do introverted students report low social engagement in their studying? Are they more prone to burnout in learning, and do they have low academic well-being (e.g., schoolwork engagement) or low general well-being (e.g., self-esteem)? How does introversion interact with social engagement and affect well-being?

### School Engagement, Social Engagement, and Introversion

School Engagement, as a multidimensional phenomenon, was conceptualized to have behavioral, emotional, and cognitive components (Fredricks et al., 2004, 2016; Rimm-Kaufman et al., 2015; Wang et al., 2016). In general, behavioral engagement refers to the manifest behaviors, such as participation or effort in academic and non-academic activities, whereas emotional engagement includes the positive affective experiences in relation to school activities. Cognitive engagement consists of mental process in school activities, such as being concentrated or using learning strategies. Some researchers also add social engagement as an important component into the framework of engagement (Appleton et al., 2006; Patrick et al., 2007; Finn and Zimmer, 2012). According to a recent study (Wang et al., 2016), social engagement consists of social interactions with peers and adults and the willingness to maintain the relationships while learning. For example, it can be engaging in discussion or listening to one's peers but also can include working cohesively, respectfully, and supporting other students' learning. Moreover, it has been argued that engagement and disengagement are related but distinct phenomenon (Wang et al., 2019). Thus, social engagement can be conceptualized as two states: social engagement and social disengagement (Wang et al., 2019). For example, it can be a self-imposed activity, interaction with other students and includes social exchange, but it can also be passive unwillingness toward collaborative learning and withdrawal from social situations. Group members can also support or undermine each other's participation in positive and negative ways: active work to support fellow group members' engagement, respecting them and working cohesively or discouraging other students from participating and disrespecting them, their statements, and their actions (Linnenbrink-Garcia et al., 2011). To reflect these social interactions, researchers created a scale to assess social engagement, which not only focuses on social–behavioral indicators but also included items that reflect social–affective (e.g., caring about others' ideas) and social–cognitive (e.g., building on others' ideas) dimensions of group interactions (Fredricks et al., 2016; Wang et al., 2016, 2019). However, so far, this social engagement scale has been only used and tested in the United States; whether the scale is a valid instrument in the context of this study, Finland, is unknown. Although both the United States and Finnish adolescents share some common western values (Davidson et al., 2008), the two countries differ in many other aspects such as population size or school system (OECD, 2020). Schools in the United States are larger, more ethnically diverse, and competition oriented than in Finland (Schneider et al., 2016). Consequently, one aim of this study is to examine the use of the social engagement scale in the Finnish school context.

Introversion, as a personality trait, refers to the individual difference in the inclinations toward the inner and outer world (Jung, 1921). Although no one is completely introverted or extraverted, usually introversion and extraversion are viewed as opposites, and introversion can also be defined as low extraversion. While extraverts like to be social with other people, introverts are more comfortable with their inner world of thoughts and feelings (Helgoe, 2010) and prefer solitude (Burger, 1995). Like all people, introverts need social relationships. However, they are selective when it comes to building social contacts, and they require more time alone to balance out their energy after social situations because they can get overstimulated (Schmeck and Lockhart, 1983). Introverts tend to be sensitive, introspective, and interested in the deeper feelings of encounters or transactions (Henjum, 1982). They are also empathetic, caring, and have good listening skills, which may enable them better to understand and help others (Cain, 2013). Two different types of introverts have also been discussed (Henjum, 1982): Type A introverts are confident, self-sufficient, and self-actualizing and can interact very well with people, whereas type B introverts are shy, lack communication skills, are timid and withdrawn, and have a low self-concept. Moreover, research indicated that introverts can have good social and group working skills (Nussbaum, 2002). In group activities, introverts work together to coconstruct solutions to problems, they listen to one another's suggestions and are less attached to their own ideas than extraverts (Nussbaum, 2002). Recent research on introversion also showed the disassociations between trait-level introversion and state-level introverted behaviors (Zelenski et al., 2013). Using the experience sampling method, a method that collects momentary self-reported experience or behavior data, researchers have observed substantial amount of behaviors that deviated from the assumed robust personality trait (Fleeson and Gallagher, 2009). Further study also found that introverts, when giving intended instructions or prompts, can act more extroverted behaviors than usual (Margolis and Lyubomirsky, 2020). Therefore, it is reasonable to expect that introversion may have a complex association with social engagement.

### Social Engagement, Introversion, and Well-Being

Given the research gap on the possible complex relationship between social engagement and introversion, we also aimed to examine the interaction of social engagement and introversion and its effects on well-being. Well-being is a broad term about personal's affective and cognitive experience and evaluation of their life (Diener, 2009; Tov, 2018). Generally, it includes three components: (1) positive affect, (2) negative affect, and (3) life satisfaction (Diener, 2009). It can be further separated on the basis of domains, such as academic well-being or general well-being (Wang et al., 2020). In this study, three well-being indicators were used: self-esteem (a representative of general well-being; Diener, 2009), schoolwork engagement, and school burnout (representatives of academic well-being; Salmela-Aro and Upadyaya, 2014; Romano et al., 2020). Self-esteem is the view on how much value people place on themselves (Baumeister et al., 2003). In school, self-esteem is important for learning, motivation, and performance (Baumeister et al., 2003). High self-esteem crates confidence in one's abilities (Epstein, 1982), and this may help a student succeed in school, although too much high self-esteem does not actually cause any improvements in academic performance (Baumeister et al., 2003). Studies showed that introverts tend to have lower self-esteem than extraverts (Bown and Richek, 1969; Tolor, 1975; Cheng and Furnham, 2003; Swickert et al., 2004). One possibility is that an introvert is more likely to withdraw in social situations, and their timid behavior gives other people an indication of low self-esteem (Lawrence, 2006). Students with low self-esteem may have a lower level of social interaction and avoid social situations because of fear of failure, and this may further threaten their self-esteem (Murberg, 2010).

Besides self-esteem, two academic well-being variables—schoolwork engagement and school burnout—were examined. In line with the work engagement literature, schoolwork engagement consists of three elements: (1) energy or vigor (e.g., high level of mental resilience while studying, positive approach to schoolwork and persistence when facing difficulties), (2) dedication (e.g., a sense of significance, perceiving schoolwork as meaningful, strong involvement in one's work), and (3) absorption (e.g., concentration and working intensively, a flow-like experience) (Schaufeli et al., 2002; Salmela-Aro and Upadyaya, 2012). Research has found schoolwork engagement to be closely related to academic performance and students' general well-being (Salmela-Aro and Upadyaya, 2012; Upadyaya and Salmela-Aro, 2013) and to have a positive association with self-esteem (Ma, 2003; Virtanen et al., 2016). School burnout is based on the theory of work burnout (Schaufeli et al., 2002) and encompasses three components: exhaustion, cynicism, and inadequacy. When students feel exhausted because of school demands, they become cynical toward studying (Schaufeli et al., 2002) and feel inadequate (Salmela-Aro et al., 2009), which diminishes their sense of competence, achievement, and accomplishment (Tuominen-Soini and Salmela-Aro, 2014; Tang et al., in press). Previous studies also have shown that school burnout is negatively associated with self-esteem (Salmela-Aro and Upadyaya, 2014) and schoolwork engagement (Salmela-Aro et al., 2009; Salmela-Aro and Upadyaya, 2014). In addition, studies found that extraversion (Storm and Rothmann, 2003; Grigorescu et al., 2018) and social support from peers (Peterson et al., 2008; Kim et al., 2018) can act as a protective factor against burnout.

### Aims of the Present Study

Given the important role that social engagement and introversion play in the student's well-being, the first aim of the present study was to examine the validity and reliability of the social engagement scale among students at Finnish comprehensive schools. The validity of social engagement was assessed by examining its associations with the participants self-reported schoolwork engagement and school burnout. The second aim of the study was to examine the interaction effect of social engagement and introversion on self-esteem, schoolwork engagement, and school burnout.

The research questions addressed in this study are the following:

Q1. Is social engagement scale a valid scale to be used in the Finnish school context? We expect that the scale can be validated for use in Finland, and it would be positively related with schoolwork engagement (Patrick et al., 2007; Fredricks et al., 2016) and self-esteem (Amirkhan et al., 1995; Murberg, 2010) and negatively with school burnout (Peterson et al., 2008; Kim et al., 2018).
Q2. How does introversion relate with self-esteem, schoolwork engagement, and school burnout? And how does social engagement's interaction with introversion affect self-esteem, schoolwork engagement, and school burnout? We expect that introversion will associate negatively with self-esteem (Bown and Richek, 1969; Tolor, 1975; Cheng and Furnham, 2003; Swickert et al., 2004; Amirazodi and Amirazodi, 2011) and schoolwork engagement (Murberg, 2010) and positively with school burnout (Peterson et al., 2008; Kim et al., 2018). We also expect social engagement to moderate the relationship between introversion and self-esteem, in such a way that introverts would be more likely to have low self-esteem if their social engagement was low. Moreover, we expect social engagement to be able to moderate the relationship between introversion and schoolwork engagement and school burnout, in such a way that introverts would be more likely to have low schoolwork engagement and high school burnout if their social engagement was low.

## Methods

### Participants and Procedure

This study is part of the Mind the Gap project (2013–2016). In total, 862 ninth grade students (age, 15–16; 59% girls) were included in the analysis. Participants were recruited on a voluntary basis. The students and their parents were contacted and informed beforehand about the study's purpose and confidential information handling. Only those who agreed to attend were included in the data collection. The data were collected in urban comprehensive schools in Southern Finland. The students were asked to complete an electronic questionnaire during the school day.

### Measures

#### Social Engagement

Social engagement scale is a subscale developed by Fredricks et al. (2016) and Wang et al. (2016). It consists of seven items measuring social engagement (e.g., “*I build on other students' ideas*,” see full list of items in Table 1). All the items are rated on 5-point Likert-type scale ranging from 1 (not like me at all) to 5 (very much like me).

**Table 1:** Descriptive and correlations of social engagement items.

**Item**	**1**	**2**	**3**	**4**	**5**	**6**	**7**
I build on other students' ideas	1.00						
I try to understand others' students' ideas in school	0.51^**^	1.00					
I try to work with students who can help me in school	0.37^**^	0.43^**^	1.00				
I try to help other students who are struggling with schoolwork	0.49^**^	0.57^**^	0.39^**^	1.00			
I don't care about other students' ideas	−0.08^*^	−0.26^**^	−0.00	−0.18^**^	1.00		
When working with other students, I don't share my ideas	−0.11^**^	−0.13^**^	−0.02	−0.06	0.49^**^	1.00	
I don't like working with my classmates	−0.08^*^	−0.18^**^	−0.02	−0.12^**^	0.52^**^	0.55^**^	1.00
Mean	2.95	3.50	3.42	3.33	2.30	2.45	2.27
SD	1.05	1.06	1.02	1.05	1.08	1.10	1.12

* *p < 0.05*,

** *p < 0.01*.

#### Introversion

The Big Five personality traits (McCrae and John, 1992) were tested by measuring extraversion, agreeableness, conscientiousness, neuroticism, openness, and sensation seeking using 21 items (Kovaleva et al., 2013). The subscale (four items) that measures extraversion/introversion (e.g., “*I am reserved*,” “*I am sometimes shy, inhibited*,” “*I am talkative*,” and “*I am outgoing, sociable*”) was used in this study. The positively worded items (last two items) were reversed to obtain a scale that measures introversion. The responses were rated on a 5-point Likert-type scale ranging from 1 (completely disagree) to 5 (completely agree). A sum score was calculated from all the four items to indicate the level of adolescents' introversion. Cronbach's α was 0.63, which is considered acceptable. The scale had good structural validity [comparative fit index (CFI) = 0.99; Tucker–Lewis index (TLI) = 0.98; root mean square error of approximation (RMSEA) = 0.05] in this study.

#### Self-Esteem

Self-esteem was measured using the Rosenberg self-esteem scale (Rosenberg, 1965). The original scale consists of 10 items, but the data used in this study consisted of five items (e.g., “*Sometimes I think I am no good at all* (reversed),” “*I wish I could respect myself more* (reversed),” “*I feel I have a number of good qualities*,” “*All in all I am satisfied with myself*,” and “*I take a positive attitude toward myself* ”) and were rated on a 7-point Likert-type scale ranging from 1 (completely disagree) to 7 (completely agree). The scale had good structural validity (CFI = 0.97; TLI = 0.92; RMSEA = 0.12) in this study. Two negative worded items were reversed, and a sum score was calculated from five items. Cronbach's α for the sum score was 0.74.

#### Schoolwork Engagement

Schoolwork engagement was assessed using the Schoolwork Engagement Inventory (Salmela-Aro and Upadyaya, 2012). This scale consists of nine items measuring energy (three items; “*At school I am bursting with energy*,” “*I feel strong and vigorous when I am studying*,” “*I feel like going to school when I get up in the morning*”), dedication (three items; “*I am enthusiastic about my studies*,” “*I find the schoolwork full of meaning and purpose*,” “*My schoolwork inspires me*”) and absorption (three items; “*Time flies when I'm studying*,” “*When I am working at school, I forget everything else around me*,” “*I feel happy when I am working intensively at school*”) in relation to schoolwork. The items are rated on a 7-point Likert-type scale ranging from 0 (never) to 6 (every day), so that a higher score indicated a higher level of engagement. For the analysis, the mean composite score of all the nine items was used to indicate overall schoolwork engagement; the scale has been validated and used intensively in the Finnish context (Salmela-Aro and Upadyaya, 2012; Tang et al., 2019). The scale also had good structural validity (one-factor model: CFI = 0.96; TLI = 0.94; RMSEA = 0.11) in this study. Cronbach's α for the sum score was 0.95.

#### School Burnout

The School Burnout Inventory (Salmela-Aro et al., 2009) is a valid instrument in Finnish contexts and consists of nine items comprising emotional exhaustion (four items; “*I feel overwhelmed by my studies*,” “*I often sleep badly because of matters related to my studies*,” “*I brood over matters related to my studies a lot during my free time*,” “*The pressure of my studies causes me problems in my close relationships with others*”), cynicism (three items; “*I feel a lack of motivation in my studies and often think of giving up*,” “*I feel that I am losing interest in my studies*,” “*I'm continually wondering whether my studies have any meaning*”) and inadequacy (two items; “*I often have feelings of inadequacy in my studies*,” “*I used to have higher expectations of my studies than I do now*”). The scale also had good structural validity (second-order factor model: CFI = 0.97; TLI = 0.94; RMSEA = 0.09) in this study. The items are rated on a 6-point Likert-type scale ranging from 1 (completely disagree) to 6 (completely agree), so that the higher score indicates a higher level of school burnout. Cronbach's α was 0.91 for the calculated sum score.

### Analysis Strategy

The statistical analyses were performed using IBM SPSS Statistics version 25 and Mplus 8.2. Confirmatory factor analysis (CFA) was conducted to test the structural validity of social engagement. The structure of social engagement scale was tested by comparing the goodness of fit of four alternative models (i.e., one-factor model, two-factor model, higher-order model, and bifactor model). Second, reliabilities (i.e., item reliability, scale reliability) of the social engagement scale were investigated. Then, the discriminant and concurrent validity of the scale was investigated by examining its associations with schoolwork engagement and school burnout, which were used as criterion validity indicators of social engagement. After testing the factor structure, the composite scores and Cronbach's alphas were calculated. The correlations were examined to determine the relations between social engagement and validity indicators (i.e., schoolwork engagement and school burnout). Finally, hierarchical multiple regression was used to test the moderator effects of social engagement on the relationship with introversion and well-being (i.e., with self-esteem, schoolwork engagement, and school burnout). Before testing the moderating effect, the predictor and moderator variables were standardized to reduce any problems related to multicollinearity between the interaction term and the main effects (Frazier et al., 2004).

## Results

### Structure and Validity of the Social Engagement Scale

**Figure A1:**
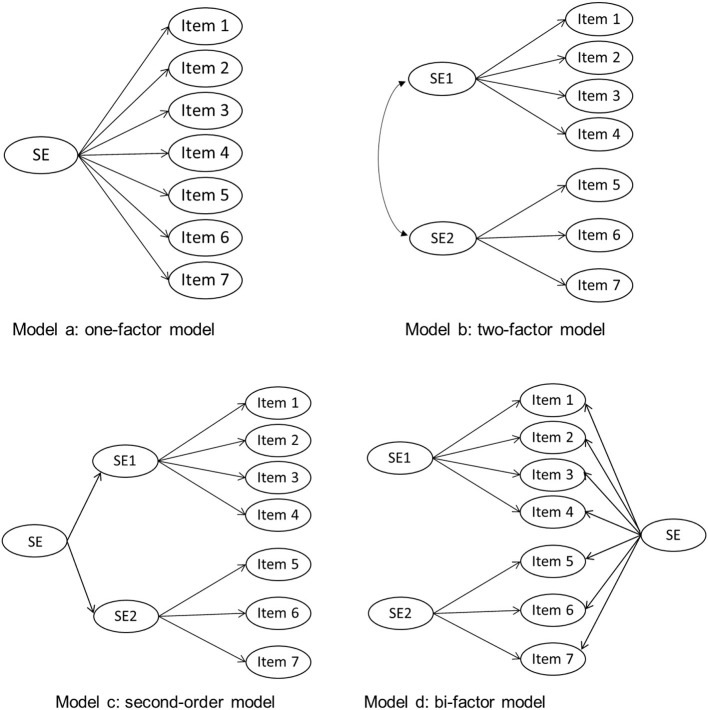
Four alternative test models for social engagement scale.

Table 1 presents a correlation table with means and standard deviations for the observed items. Items 1–4 were positively correlated (>0.30) and items 5–7 negatively correlated. As all the items were normally distributed, confirmatory factor analysis was used to determine the structure of the social engagement. Four alternative models (see Figure A1) were estimated separately: (1) a one-factor model, namely SE; (2) a two-factor model that assumed two correlated latent factors, namely, SE1 and SE2; (3) a second-order model placing SE1 and SE2 as first-order factors and SE as the second-order factor, which explained all covariance among first-order factors; and (4) a bifactor model that estimated SE as another general factor in addition to SE1 and SE2.

**Figure 1:**
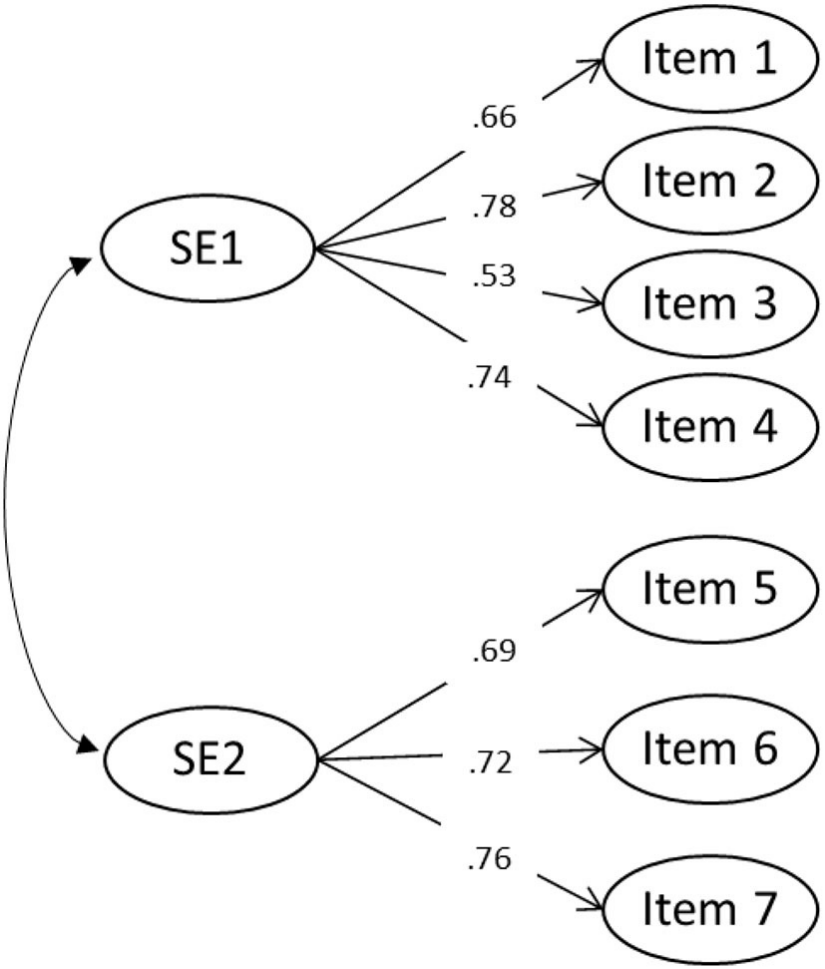
Final two-factor model for social engagement scale.

The one-factor model did not have a good fit, χ^2^(14) = 499.82, *p* < 0.001; CFI = 0.51; TLI = 0.27; RMSEA = 0.21; standardized root mean square residual (SRMR) = 0.14. The two-factor model had a good model fit for social engagement, χ^2^(13) = 48.54, *p* < 0.001; CFI = 0.96; TLI = 0.94; RMSEA = 0.06; SRMR = 0.04, thus superior to the one-factor model. Either the second-order model^1^ or the bifactor model could be identified, which thus yielded null model estimates. This may be due to the scale having only two factors and the testing of complex models being problematic without additional constraints. Therefore, the two-factor model was chosen as the best model. Figure 1 presents the standardized validity coefficients (i.e., factor loadings) obtained.

After confirming the scale structural validity, the composite scores were calculated accordingly, as well as Cronbach's alphas. The items that clustered on the same factor were named SE1 = Social Engagement (α = 0.77) and SE2 = Social Disengagement (α = 0.77). The social engagement and social disengagement correlated negatively (*r* = −0.18, *p* < 0.01; see Table 2). Next, the associations between social engagement, schoolwork engagement, and school burnout were examined to investigate the criterion validity of the social engagement scale. The results (see Table 2) showed that the social engagement correlated positively with schoolwork engagement (*r* = 0.41, *p* < 0.01) but not school burnout (*r* = 0.02, *p* < 0.10). Social disengagement correlated negatively with schoolwork engagement (*r* = −0.10, *p* < 0.01) and positively with school burnout (*r* = 0.26, *p* < 0.01).

**Table 2:** Descriptive and correlations for study variables.

	**1**	**2**	**3**	**4**	**5**	**6**
1. Social engagement	1.00					
2. Social disengagement	−0.18^**^	1.00				
3. Introversion	−0.13^**^	0.26^**^	1.00			
4. Self-esteem	0.12^**^	−0.22^**^	−0.31^**^	1.00		
5. Schoolwork engagement	0.41^**^	−0.10^**^	−0.05	0.22^**^	1.00	
6. Burnout	0.02	0.26^**^	0.14^**^	−0.40^**^	−0.19^**^	1.00
*N*	824	823	766	765	862	858
*Mean*	3.30	2.34	2.83	4.55	4.33	2.83
*SD*	0.81	0.91	0.82	1.20	1.48	1.14
*Item range measured*	1–5	1–5	1–5	1–7	1–7	1–6

** *p < 0.01*.

### Interaction Between Social Engagement and Introversion and Its Role in Self-Esteem, Schoolwork Engagement, and School Burnout

The second aim of this study was to examine the effect of the interaction between social engagement and introversion on self-esteem, schoolwork engagement, and school burnout. As a preliminary step, descriptive statistics and correlations were obtained from all the variables (Table 2). Introversion correlated negatively with the social engagement (*r* = −0.13, *p* < 0.01) and positively with social disengagement (*r* = 0.26, *p* < 0.01). It correlated positively with school burnout (*r* = 0.14, *p* < 0.01) and negatively with self-esteem (*r* = −0.31, *p* < 0.01). The correlation between introversion and schoolwork engagement was not significant (*p* > 0.05). The social engagement correlated positively with schoolwork engagement (*r* = 0.41, *p* < 0.01) and self-esteem (*r* = 0.12, *p* < 0.01). The correlation with school burnout was not significant (*p* > 0.05). Social disengagement correlated negatively with schoolwork engagement (*r* = −0.10, *p* < 0.01) and self-esteem (*r* = −0.22, *p* < 0.01) and positively with school burnout (*r* = 0.26, *p* < 0.01). Only self-esteem correlated significantly with the social engagement, social disengagement, and introversion, and thus self-esteem was selected for further analysis.

To test whether social engagement moderates the relationship between introversion and well-being (i.e., self-esteem, schoolwork engagement, and school burnout), a hierarchical multiple regression analysis was conducted. In the first step, three variables were included: the social engagement, social disengagement, and introversion. Next, the interaction term between the social engagement/social disengagement and introversion was added to the regression model (Table 3).

**Table 3:** Role of introversion, social engagement scale, and their interactions on well-being.

	**Self-esteem**		**Schoolwork engagement**		**School burnout**	
	**B**	**β**	**B**	**β**	**B**	**β**
**Step 1**						
Social engagement (SE)	0.06	0.06	0.40^**^	0.41^**^	0.06	0.06
Social disengagement (SDE)	−0.14^**^	−0.14^**^	−0.03	−0.03	0.24^**^	0.25^**^
Introversion	−0.27^**^	−0.27^**^	0.01	0.01	0.08^*^	0.08^*^
**Step 2**						
SE x Introversion	0.08^*^	0.09^*^	0.02	0.02	−0.06	0.07
SDE x Introversion	0.02	0.02	−0.01	−0.01	−0.05	−0.05

* *p < 0.05*,

** *p < 0.01*.

**Figure 2:**
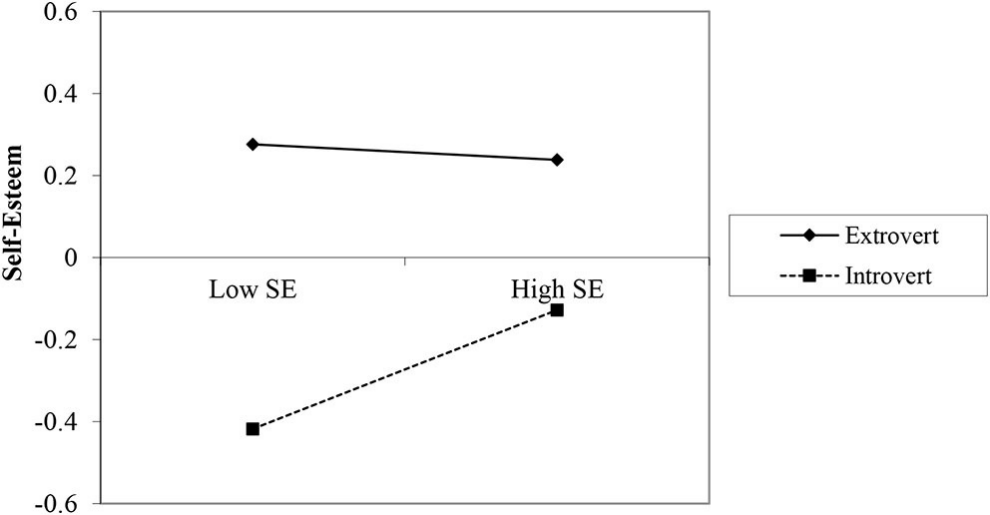
Interaction of social engagement (SE) and introversion on self-esteem.

In first step, the variables accounted for a significant amount of variance in self-esteem [R^2^ = 0.126, *F*_(3, 714)_ = 34.24, *p* < 0.001]. In the second step, the interaction term accounted for a significant proportion of the variance in self-esteem [ΔR^2^ = 0.007, Δ*F*_(2, 712)_ = 2.99, *p* = 0.05]. The results revealed no significant positive relation between the social engagement and self-esteem (B = 0.06, *p* > 0.05), but there was a significant negative relation between the social disengagement and self-esteem (B = −0.14, *p* < 0.001) as well as between introversion and self-esteem (B = −0.27, *p* < 0.001). The unstandardized regression coefficient for the interaction term for the social engagement and introversion was significant (B = 0.08, *p* < 0.05; Figure 2) and not significant for social disengagement and introversion (B = 0.02, *p* > 0.05). Examination of the interaction plot revealed that introverts with high social engagement have higher self-esteem than introverts with low social engagement. However, high or low social engagement had no effect on self-esteem among extraverts.

For schoolwork engagement and school burnout (see Table 3), no statistically significant interaction effect could be found for introversion-social engagement or introversion-social disengagement. However, some main effects could be found; for instance, social engagement showed positive association with schoolwork engagement (B = 0.40, *p* < 0.001). In contrast, social disengagement (B = 0.24, *p* < 0.001) and introversion (B = 0.08, *p* < 0.05) were associated positively with school burnout.

## Discussion

### Findings and Discussion

The aim of this study was to examine the validity and reliability of the social engagement scale among students in Finnish comprehensive schools. Before this study, the social engagement scale had only been used in the United States and for examining learning in math and science classes (Rimm-Kaufman et al., 2015; Fredricks et al., 2016; Wang et al., 2016). This study revealed that the social engagement scale is a valid measure that can be used in the Finnish school context.

The examination of the structure of the scale indicated that a two-factor model best fit the social engagement scale. This model suggests that students' social engagement is characterized by two unique dimensions, which were named the social engagement and social disengagement. The social engagement indicates willingness for collaborative learning and helping peers, whereas social disengagement indicates unwillingness to work with peers and share ideas. Statistical support for the validity of the social engagement scale was found when each factor loaded clearly onto two factors and there was no cross-loading, suggesting that each factor assessed the unique variance attributed to the engagement or disengagement subtype. We also found that social engagement and disengagement associated with schoolwork engagement and school burnout in a different pattern. Social engagement correlated positively with schoolwork engagement but not school burnout; however, social disengagement correlated negatively with schoolwork engagement and positively with school burnout. The results of this study indicate that social engagement and disengagement play important but different roles in learning. As it has been stated earlier (Patrick et al., 2007; Fredricks et al., 2016), the social engagement should be endorsed to motivate students' engagement in school. On the other hand, social disengagement associated with the risk of burnout (Storm and Rothmann, 2003; Grigorescu et al., 2018). This finding is also in line with the recent discussion about the engagement–disengagement disassociation (Wang et al., 2019). Although many studies have approached engagement as the opposite of disengagement, an increasing number of studies have suggested that disengagement should be perceived as a separate and distinct psychological process that makes a unique contribution to academic learning (Wang and Degol, 2014; Skinner, 2016; Salmela-Aro et al., 2017). For example, studies applying a person-oriented approach showed that engagement coexisted with high exhaustion and amotivation (Tuominen-Soini and Salmela-Aro, 2014; Salmela-Aro et al., 2016). These findings thus imply that disengagement is not simply the opposite of engagement but a distinct psychological process that contributes independently to academic and psychological outcomes: a student can be engaged and disengaged at the same time.

The second aim of this study was to examine the interaction effect of social engagement and introversion on self-esteem, schoolwork engagement, and school burnout. Because introverted people often choose to be by themselves, it was worthwhile examining how social engagement and introversion would interact. The results demonstrated that the interaction between the social engagement and introversion was significant: introverts with high social engagement have higher self-esteem than introverts with low social engagement, which supports previous research (Schmidt and Fox, 1995; Nussbaum, 2002). This may indicate that, for all students, no matter what their personality trait is, it is important to collaborate with other students and to have opportunities to share ideas with them and receive help from them when needed. However, it is important to note that social engagement explained about 13% of the total effect, which means that other unexplored variables may affect self-esteem.

Interaction terms for the social engagement and social disengagement for schoolwork engagement and school burnout were not found. This may be because schoolwork engagement and school burnout measure academic well-being, whereas self-esteem measures general well-being. Although social engagement correlated with schoolwork engagement and school burnout, it seems that neither being socially engaged nor being socially not engaged affected this. This may indicate that there are different operators behind academic well-being and general well-being. However, it is important to note that, as results in this study revealed, the social engagement has a high positive relation with schoolwork engagement and social disengagement has a negative relation with schoolwork engagement and a positive relation with school burnout. This means that, regardless of personality type, having a high social engagement means high schoolwork engagement. However, high social disengagement decreases schoolwork engagement and raises the risk of burnout in school. Nevertheless, the findings revealed that one way in which to improve introverted students' well-being is to make them socially engaged. To be socially engaged one needs to have good social skills and be socially competent. School is usually the place in which to learn these skills. This requires a socially supportive environment in which students feel that they belong; they have to be accepted by teachers and peers and must have opportunities to interact with both.

Our results indicated that introversion and social disengagement have a positive relation and that introverted students with low social engagement do not help their peers, are not interested in other students' ideas, and do not share their own ideas. However, the study also indicated that introverts are not necessarily unsocial and that many of them are socially engaged. In Western cultures, extraversion seems to be more socially preferable and introversion less desirable (Myers, 1992). The findings in this study indicate that, for introverted people, it is useful to communicate with and be interested in others. If introverted students lack social skills, they should be taught such skills to enable them to work with each other. Introverted students should be encouraged to work with other students: Even though they do not like too much noise and do not want to be the center of attention, it would be useful for them to have different ways of interacting with other students. Extraverts and introverts both enjoy interacting with others, but extraverts do so more frequently (Srivastava et al., 2008). Another researcher also found that introversion and passive behavior (not participating in school and non-interactivity with others) have a significant positive relationship (Murberg, 2010). The introverted students reported less perceived support from fellow students than the more active students. This means that extraverts might have more opportunities to seek out and receive support from others than introverts and that support maybe not be so readily available to all students. Social skills help extraverts communicate with others and receive positive feedback, which in turn may encourage them to engage more in social activities (Cheng and Furnham, 2003), whereas introverts may feel insecure and lack acceptance (Murberg, 2010). Introverted students can feel threatened if they need to share their ideas in front of the whole class because they do not want to discuss ideas straightaway. This is why they need time to gather their thoughts before sharing them. Teachers should arrange their classrooms to be encouraging of interaction with other students. Quiet places to work and opportunities to work in small and familiar groups would help introverted students participate more. This would give them positive social experiences, and they would not feel so threatened in social situations that may promote more active behavior. This may raise their self-esteem and encourage them to socially commit even more. Less importance should be placed on students participating in class discussion because this may shut some of them down.

In addition, our findings highlight the importance of identifying, understanding, and accepting different personalities at school. Teachers should identify and talk with their students about different personalities because this helps teachers identify students' needs for support and helps students respect different personalities in classes. Finally, our results are important in order to boost introverted students' self-esteem and through this to improve their well-being. However, as it has been described earlier (Baumeister et al., 2003), efforts to boost self-esteem will not necessarily foster improved outcomes and can lead to less desirable consequences, such as narcissism. They recommend using praise as a reward for socially desirable behavior and self-improvement to boost self-esteem. This recommendation justifies the usage of self-esteem in this study because, today, acting socially at school is approved of and leads to many other advantages in life.

## Conclusion

This study shows that the social engagement scale is a valid measure for the Finnish school context. More importantly, it reveals that social engagement plays an important role in introverts' self-esteem. Although, in general, introverts tend to have low social engagement, the results of this study show that they can have high social engagement in their learning, and once they are able to join groups and enjoy teamwork, their self-esteem can grow. Higher self-esteem is not necessarily better, as former studies have found (Baumeister et al., 2003), but this does not mean that it should be ignored. Low self-esteem has detrimental effects on learning and motivation (Baumeister et al., 2003), and this study show that by encouraging and ensuring that introverts engage with their peers in learning, their risk of low self-esteem can decrease and they can enjoy the same level of self-esteem as their extraverted peers. These findings also remind teachers to take their students' personalities into consideration and encourage introverted students to engage more in peer learning.

### Limitations and Future Direction

It is important that the findings of this study be interpreted in the light of the following limitations. First, current study is a cross-sectional study. Thus, no cause–effect can be concluded from our results. Second, in this study, all the data were self-reported by students, which inevitably creates a few limitations. One is that people tend to answer questions in a manner that others will view favorably (social desirability; Edwards, 1957), and this seems to be the case in personality inventories (Bäckström et al., 2009) and self-esteem (Baumeister et al., 2003). Self-reported measures may produce measurement errors because factors other than those being measured will influence how people respond (Field, 2013). This is also the case in measuring introversion, as extraverted people tend to report experiencing more positive emotions, whereas introverts tend to be more neutral (Myers, 1992). Thus, it is possible that the students in this study did not answer the questionnaires completely honestly, and the number of introverts in the data may actually be even higher. Adolescents are in the process of building their self-esteem, and how they define and evaluate themselves is complex. Peer-reported personality could improve the internal reliability of the Big Five questionnaires (McCrae and Terracciano, 2005), and this should be considered in future research. Third, variables from family such as parents' marital status or sibling situations were not considered in the study. Family environments can impact the formation of personality (e.g., introversion) and school engagement (Fredricks et al., 2004; Cain, 2013; Zelenski et al., 2013); however, they were not taken into account in this study, as the project mainly focused on individual-level variables. Future studies may include family-level variables in their examinations with introversion and social engagement. Finally, this study took a variable-centered approach and described the associations between introversion, social engagement and well-being. In the future, it may be worthwhile also taking person-oriented approach to identify groups of individuals who share particular attributes or relations among attributes. In this way, groups that need most support could be identified properly. For example, having a high social engagement and low introversion could have a different impact on student outcomes such as well-being than high social disengagement and high introversion.

## Data Availability Statement

The raw data supporting the conclusions of this article will be made available by the authors, without undue reservation.

## Ethics Statement

The studies involving human participants were reviewed and approved by Faculty of Educational Sciences, University of Helsinki. Written informed consent to participate in this study was provided by the participants' legal guardian/next of kin.

## Author Contributions

ST and XT conceptually designed the study, carried out analyses, interpreted the results, and drafted and revised the manuscript. KS-A conceived the research project, curated the research data, and reviewed and revised drafts of the manuscript. All authors read and approved the final manuscript.

## Conflict of Interest

The authors declare that the research was conducted in the absence of any commercial or financial relationships that could be construed as a potential conflict of interest.

## Footnotes

^1^The second-order model was identified once we fixed the residual variance of the Social Disengagement factor to zero. However, the model fits then were the same as the two-factor model's fits given that the two models start to become mathematically identical after the fixation.
